# Recommendations to mitigate barriers to uptake and delivery of a four-dose malaria vaccine schedule: insights from the MVIP’s qualitative evidence

**DOI:** 10.1186/s12936-025-05611-3

**Published:** 2025-11-18

**Authors:** Jessica Price, Yvette Collymore, John Tanko Bawa, Rouden Esau Mkisi, Cayenne Buell, Rose E. Jalang’o, Mike Chisema, Kwame Amponsa-Achiano, W. Scott Gordon

**Affiliations:** 1 PATH, 437 N 34th Street, Seattle, WA USA; 2 Ministry of Health, Kenya, National Vaccines and Immunization Programme, Nairobi, Kenya; 3 Ministry of Health, Malawi, Expanded Programme on Immunization, Lilongwe, Malawi; 4 Ghana Health Service, Expanded Programme on Immunization, Accra, Ghana

**Keywords:** Malaria, Malaria prevention, Immunization, Vaccine, Vaccine access, Vaccine uptake, Vaccine acceptance, Vaccine demand

## Abstract

**Background:**

Malaria vaccination was piloted in selected areas of three countries to produce evidence that would inform global recommendations on the public health use of the RTS,S/AS01_E_ (RTS,S) malaria vaccine and to inform guidance for countries planning to introduce the vaccine into childhood immunization systems. This paper focuses on challenges to the uptake and delivery of a four-dose RTS,S schedule, presents actionable insights into qualitative data from the 2019–2023 Malaria Vaccine Pilot Evaluation in Ghana, Kenya, and Malawi, and presents recommendations to address barriers to vaccine uptake.

**Methods:**

This paper draws on published qualitative data from the three pilot countries and unpublished expert analysis. It draws on findings previously reported in publications from a qualitative longitudinal study, the Healthcare Utilization Study (HUS), conducted in a subsample of communities where RTS,S was introduced during the pilots. The study elicited perceptions from caregivers of eligible children about the vaccine alongside other relevant topics, such as their use of vaccination services. Interviews with health workers focused on their evolving perceptions about the vaccine and experiences integrating its delivery into routine immunization. Technical experts involved in the vaccine pilot and implementation managers from the pilot countries provided insights from their experiences. This feedback helped validate both the findings and the potential practicality of recommendations for scaling up delivery.

**Results:**

Despite good acceptance of the vaccine, many children missed some or all doses due to overlapping challenges to acceptance and delivery of the four-dose schedule. Highlighting the barriers to uptake, the paper discusses guidance, tools, and materials that may help programme staff develop strategies, plans, and materials, including strategies to address the complexity of the schedule and to track four doses for individual children.

**Conclusions:**

Adding a four-dose vaccine to a crowded routine immunization schedule presents challenges, as seen during the pilots. The malaria vaccine experience and several studies have provided guidance and tools to support malaria vaccine expansion in the three countries and introduction of the vaccine elsewhere. The observations and recommendations in this paper are intended as a complement to the wide range of materials, guidance and tools already available.

## Background

The intent of the Malaria Vaccine Implementation Programme (MVIP) was to pilot malaria vaccination in selected areas of three countries’ routine immunization systems and produce evidence that would inform global recommendations on the public health use of the RTS,S/AS01_E_ (RTS,S) malaria vaccine [1, 2]. The MVIP was also well placed to inform the development of guidance for countries planning to introduce the vaccine [3]. To this end, as described in the papers included in this collection, the Malaria Vaccine Pilot Evaluation used a wide range of research methods and data types to determine the effectiveness of RTS,S in reducing childhood deaths; to establish the feasibility of providing a four-dose schedule through routine immunization services; to consolidate information about safety in children who received the malaria vaccine; and to assess acceptability of the vaccine. The vaccination schedule requires additional injections and new vaccination visits as well as the continuation of other malaria prevention behaviours [4–6]. The MVIP’s focus on *effectiveness*, *feasibility*, *safety*, and *acceptability* of the vaccine also reflects the need to promote evidence-informed public health practice [7–9]. In addition to asking relevant questions and acquiring and appraising the evidence, actions to apply the findings to improve health outcomes are key to completing the research-to-practice process [10].

This paper is a key component of the pilot evaluation as it presents actionable insights into the qualitative data. The paper aims to advance ongoing efforts, by focusing on qualitative findings about barriers to the uptake and the delivery of a four-dose RTS,S schedule. For example, on acceptability and feasibility of the four-dose schedule, a longitudinal qualitative study was conducted to address *why*, *what*, and *how* questions [11], such as the following: *Why* do child caregivers refuse, accept, and adhere or not adhere to the four-dose schedule? *What* service delivery challenges impeded or facilitated completion of the schedule? *How* did barriers and challenges resolve or persist over time, and *how* would they be averted or mitigated in malaria vaccine expansion efforts?

The overall MVIP design, involving data collection in real world contexts (i.e., where the malaria vaccine was being delivered through routine immunization services) and in diverse community and clinic settings across the three MVIP countries, should help to ensure the broad relevance and applicability of the findings. This publication focuses on seven main reasons children received fewer than four malaria vaccine doses during the MVIP, a topic that is highly pertinent for both new malaria vaccine introductions and for programme improvement in countries where the vaccine is already being provided. The paper is structured around each of the seven reasons for missed doses, briefly describing top-level findings and then elaborating on possible programmatic actions to address the barriers identified. To promote evidence-linked action, the paper highlights guidance, tools, and materials that may help programme staff to develop implementation strategies, plans, and materials.

## Sources of qualitative evidence

The MVIP’s qualitative longitudinal study, the Healthcare Utilization Study (HUS), was conducted in a subsample of communities where RTS,S was introduced as part of the MVIP [12]. For purposes of this study, a community was defined as the smallest administrative unit served by at least one health facility. For a total of 27 communities included in the HUS, nine communities were purposefully selected in each MVIP country to achieve maximum variation [13] in socio-demographic characteristics and in vaccination coverage (as obtained from data from national immunization programmes). Palinkas et al*.* [13] propose that purposeful sampling in qualitative studies is a way “to obtain cases that are information-rich,” meaning inclusion of participants with specific knowledge or experiences relevant to the study. For this paper, such participants included caregivers of children eligible to receive the malaria vaccine and health workers who were delivering the vaccine as part of the MVIP.

From the 27 communities, a cohort of 198 caregivers of children eligible to receive the malaria vaccine were selected to participate in three individual interviews over the 18-month RTS,S immunization schedule. The study selected sites and enrolled participants to achieve comparability across sites, individuals, and countries. Round 1 (R1) interviews occurred soon after the vaccine was first introduced in the community and when the caregiver’s child would have been eligible to receive dose one (RTS,S-1); Round 2 (R2) interviews were done mid-way through the study period when the child should have received three RTS,S doses (RTS,S-3), at around 12 months of age. Round 3 (R3) interviews were conducted when the child was around 24-months old and when the child should have received the final RTS,S dose (RTS,S-4).

Across the three interview rounds, the same qualitative interview guide elicited caregivers’ evolving perceptions about vaccines alongside other relevant topics, such as their utilization of vaccination services. Also at each interview round, the child’s vaccination history was obtained from the child’s health card to determine malaria vaccine uptake by the end of the study. Vaccination data were treated as missing if the child’s health card could not be located. Price et al*.* [12] provide a full description of the methods used for data collection with caregivers. Price et al. [12] and Gurley et al. pers. commun. present findings from caregiver interviews across the three countries, the former focusing on uptake of the four-dose schedule and the latter describing the effects of vaccine uptake on continued use of insecticide-treated nets (ITNs) and on malaria treatment seeking in instances of fever or when malaria was suspected by the caregiver.

In the same 27 communities where child caregivers were sampled, health workers responsible for administering childhood vaccinations at the primary health care level were enrolled in the study. The same interview guide was used across the three countries, but small-group or individual interviews were done with health workers according to the country’s research team’s preference. During R3, 49 interviews (36 individual and 13 small group) were conducted with health workers. Interviews with health workers focused on their evolving perceptions about the RTS,S vaccine, experiences integrating its delivery in routine immunization services, and service delivery challenges encountered. Coding was done, based on these themes of health worker perceptions from all three MVIP countries to identify the main issues affecting uptake and delivery of the four-dose schedule. In a comprehensive review of health system perspectives on the integration of the vaccine into the immunization program in Kenya, Hill et al. [14] detail methods and findings of interviews with health personnel in Kenya. After each round of HUS data collection, cross-country and country-specific theme-based analyses were done to establish preliminary findings and to observe important changes over time.

This paper draws on findings previously reported in publications and described in round-specific HUS reports. The paper also draws on unpublished analysis from technical experts involved in the vaccine pilot and implementation managers from the pilot countries who provided insights from their experiences with the MVIP. The mix of co-authors reflects essential elements of the “evidence ecosystem” [15], including people engaged in aspects of research design, conduct, and analysis (JP, CB, and WSG), technical experts (YC, JTB, REM, RNW, WSG), and Expanded Programme for Immunization managers (REJ, MC, KAA) from Ghana, Kenya, and Malawi. Feedback from the technical experts helped validate the findings and confirm the potential value and practicality of the recommendations for scaling up malaria vaccine delivery in the MVIP countries.

In October 2023, the World Health Organization (WHO) approved a second vaccine, R21/Matrix-M, for the prevention of malaria in children [16]. It is expected that the evidence and recommendations discussed in this paper are applicable to R21/Matrix-M, given its similarity both to the RTS,S schedule and mode of delivery [17]. In the remainder of this paper, the “malaria vaccine” is used as a generic reference to one, either, or both R21/Matrix-M and RTS,S vaccines.

## Why did children receive fewer than four malaria vaccine doses?

Despite some caregivers’ initial reluctance to accept a new vaccine and more injections, acceptance of the first three vaccine doses in the HUS sample was good [12], paralleling findings from the MVIP’s household survey [18]. The child caregivers interviewed for the HUS expressed high trust in child health services overall and believed vaccines to be safe and needed. After initial malaria vaccine doses were given, caregivers in the HUS sample overwhelmingly perceived reduced frequency of clinical and severe malaria in their own children and in the community, further enhancing their confidence in the vaccine. Nonetheless, many children during the MVIP missed some or all doses due to diverse, but often overlapping, barriers and challenges to acceptance and delivery of the four-dose schedule.

The main barriers identified in the study can be grouped as follows:

1. Communication gaps and needs
2. Complexity of the schedule
3. Challenges tracking doses for individual children
4. Fourth-dose complacency
5. Subnational implementation
6. New vaccine hesitancy
7. Service interruptions

Each of these barriers is reviewed below, followed by specific recommendations to address them.

## Barrier #1: Communication gaps and needs

Whether a demand- or supply-side issue, most of the reasons malaria vaccine doses were missed had a communication and demand promotion element, typically betraying a critical need for more information, reassurances, or support. In this sense, unmet communication, education, and outreach need was an overarching reason malaria vaccine doses were missed during the MVIP. This meant that in some cases, caregivers were unaware of the number of doses required and of when to return for the next dose.

In all three MVIP countries, caregivers cited health workers at under-five clinics as the main source of malaria vaccine information. Community-based outreach, radio, television, and other media were also cited but far less frequently. Group health talks by health workers were essential for making announcements, providing reminders, and conveying information about the malaria vaccine’s safety, dose schedule, and need to continue using bed nets. However, delivery of and participation in group health talks during the MVIP were inconsistent (Box 1). In Malawi, where the vaccine was launched in a ‘silent introduction’ (i.e., no high-visibility official ceremony with political, community, or other leaders), missed doses due to information gaps were initially more frequent and persistent than in Ghana and Kenya [12].

**Table:** 

Box 1: Limitations of Group Health Talks.	
Group health talks at under-five clinics are essential for new vaccine introductions. However, they alone are not enough to reach all caregivers or to meet their different information needs	
Due to staff shortages, health talks are sometimes not given or are cursory in nature—"*They didn’t go deep in their explanations”.* Having their own time constraints, many child caregivers intentionally arrive late (“*I never go early”*), skipping the health talk but arriving in time to receive the service. Other caregivers describe being unable to hear the health talk over the clatter of a busy clinic	
Despite having questions, many caregivers will not ask questions at group health talks, most often out of fear of being ridiculed (“*If I don’t ask a good question, they may laugh at me*”) or criticized (“*If you ask questions, others complain that you’re slowing the services down*”)	

Health workers were aware of the limitations of the health talks. When prompted about support needed to enhance malaria vaccine education and communication, health workers emphasized facility-based needs (e.g., “client-friendly education materials”, “PA systems so they [clients] can hear”, and “audio-visual equipment” to project educational messages in the waiting area) and broader, community-based needs (e.g., more “opinion” and community leader” engagement, high-visibility visits from MOH “superiors,” and community campaigns with “information vans”). Importantly, sustaining public announcements and malaria vaccine messaging after the introduction was also seen as an important source of support for service delivery.

The qualitative data additionally reveals communication and messaging gaps specific to each of the barriers discussed in subsequent sections; this paper describes these specific communication gaps in their respective sections. The recommendations offered below are general in nature and crosscut the specific needs in relation to Barriers 2 through 7.

### Recommendations to address communication gaps and needs (Barrier 1)

#### Communicate to vaccinate—Adopt or reinforce a systematic and comprehensive approach to vaccination communication

Kaufman and colleagues [19] offer a valuable and practical approach for planning and implementing comprehensive communication strategies to promote demand for vaccines. They propose a ‘Communicate to Vaccinate’ (COMMVAC) framework that (i) identifies seven purposes of vaccination communication (Table 1); (ii) specifies types of interventions for each purpose, providing examples of intervention types; and (iii) calls out complementary aspects of communication within routine immunization contexts and vaccine campaigns. All the communication gaps and needs identified in the MVIP qualitative data are well reflected in COMMVAC framework.

**Table 1:** “Communicate to Vaccinate” framework

Purpose	Intervention types and examples
*Inform or Educate* Interventions to enable people to understand the meaning and relevance of vaccination to their health and the health of their family or community. Interventions may be tailored to populations and can also serve to address misinformation	Interpersonal communication (face-to-face interactions, one-on-one or in groups)
	Printed material (pamphlets, brochures, fact sheets, media kits)
	Phone (telephone calls, hotlines or SMS)
	Objects, devices or tools (vaccination cards, t-shirts)
	Web-based (social media, websites)
	Community event (outreach, campaigns, high-profile events)
	Edutainment (song, skit, docudrama)
	Mass media (announcements and messages delivered through newspapers, radio, TV, town criers)
	Influential spokespeople (messages delivered by recognizable or influential people)
*Remind or Recall* Interventions to remind consumers of required vaccinations and to recall those who are overdue	Interpersonal communication (face to face interactions, one-on-one or in groups)
	Phone (telephone calls, hotlines or SMS)
	Objects, devices or tools (vaccination cards)
	Community-based outreach (community health worker reminders, community announcements)
	Electronic or physical prompts for providers (reminders targeting healthcare providers during consultations)
*Enhance Community Ownership* Interventions to increase community participation and promote interaction between the community and health services. They may build community trust of vaccination, enhance involvement in planning, delivery, research, mobilization, advocacy or governance	Community involvement (input in planning or research and engagement of community health workers, mothers' support groups, social mobilisers)
	Engagement of local opinion leaders (faith leaders, local government officials, community leaders)
	Partnership building (vaccine organizers forming partnerships with local businesses, religious centers, community organizations)
	Community coalition (community or district health committees)
*Teach Skills* Interventions focusing on the acquisition of skills related to accessing vaccination services and communicating about vaccination	Communication training (training for spokespersons, lay health workers, and health professionals)
	Child caregiver skills (support to find, access, and utilize vaccination services; education on content and use of the child health card)
*Provide Support* Interventions, often tailored or personalized, to assist people in addressing specific challenges to vaccination that arise within their day-to-day lives	Interpersonal communication (face-to-face interactions, one-on-one or in groups)
	Phone (telephone calls, hotlines or SMS)
	Home visits (by health professional or lay worker)
*Facilitate Decision-Making* Interventions that extend beyond informing or educating to explaining evidence-based information about the risks and benefits; helping people consider their personal values and options related to the decision to vaccinate their child	Interpersonal communication (face-to-face interactions, one-on-one or in groups)
	Phone (telephone calls, hotlines or SMS)
	Home visits (by health professional or lay worker)
*Enable Communication* Interventions that purposefully aim to bridge a communication gap/make communication possible with particular people or groups. This may include translation beyond routine practice in a particular setting, such as translation into local or minority languages, adaptation of materials for a low- or no-literacy population, translation into Braille, or the use of interpreters	Interpreters (translation into local languages, adaptation of materials for a low- or no-literacy population, signing, and Braille)

Adapted from Kaufman et al. [19]

The MVIP countries were well prepared for the launch of the malaria vaccine, inclusive of communication and training plans with clear messaging targeting child caregivers and health workers (Table 2). Despite this preparedness, all three countries were challenged by the complex and multi-faceted communication demands to support the vaccine’s launch and successful delivery through routine immunization services.

**Table 2:** Key messages for health workers to deliver to caregivers about malaria vaccination (for country adaptation) with “Triple A” communication: Advise–Alert–Arrange [3]

Advise	
Advise on the malaria vaccine and schedule The malaria vaccine is safe and effective For the best protection a child should receive all four vaccine doses: A child receives the first dose from [X] months of age. If the child presents late, the first dose may be given up to X months of age The recommended schedule is [X] months, [X] months, [X] months and [X] months Remind caregivers that the child will need a fourth dose at around [X] months to prolong protection. If the child presents late, the fourth dose may be given up to X months of age	
Children who come late for doses should still receive their vaccination The minimum period between doses is 4 weeks The malaria vaccine is initially being introduced here as part of a phased introduction because children in this area are at high risk of getting malaria. Vaccine delivery will expand to other areas as supply increases. [IF APPLICABLE]	
Advise on other vaccinations and health services that are due (*Check the child’s home-based record; inform caregivers of other health services that are due, such as: deworming, growth monitoring, vitamin A, and other vaccinations.*)	

**Alert**
Alert on side effects
Common side effects are fever, irritability, and injection site pain and swelling (*Remind caregivers to return to the nearest health facility if they notice any side effects*.)
Alert on malaria prevention
The vaccine is part of a recommended malaria prevention package that includes other preventive measures such as insecticide treated nets, perennial or seasonal malaria chemoprevention, and indoor residual spraying [for country adaptation]

Arrange
Arrange for the next visit to ensure completion of four-dose schedule
(*Write on the home-based record the date of the next visit to receive the malaria vaccine along with other vaccines and child health services according to the schedule*.)

Considering this experience from the MVIP, an overarching recommendation for malaria vaccine expansion is to adopt or reinforce a systematic and comprehensive approach to vaccination communication, such as COMMVAC [19]. Taking such an approach can help to anticipate, preempt, and respond effectively to information and education needs, including the barriers to four-dose completion that are discussed in subsequent sections. Comprehensive malaria vaccination communication would include interpersonal communication training for health workers, community engagement strategies, relationship building with stakeholders and media, and key messages adapted for local and country contexts (Table 1). It is critical that key messages are consistently delivered, reinforced through multiple channels, and supplemented with personalized messaging in cases of high refusal or default risk. Supplemental messaging is required to respond to unique situations and barriers to uptake and dose completion, as described below.

## Announce the malaria vaccine with a *wide* and *loud* launch

Launching the malaria vaccine with a high visibility public event is an essential first step to setting the stage for successful introduction. In addition to serving the purpose of informing and educating communities (Table 1) about the vaccine, as appropriate, early announcements can also include explanations about why the country is introducing the vaccine sub-nationally. To minimize misconceptions and fears of favoritism (see Barrier 5), public announcements can describe the rationale used to select locations where the vaccine will initially be provided, the process used to select these locations, including stakeholder involvement, and the country’s plans and timelines to phase in malaria vaccination at other locations can also be communicated.

## Barrier #2: Complexity of the schedule

Providing and tracking a four-dose schedule that requires new vaccination visits was a key challenge to integrating the malaria vaccine into routine immunization services through the MVIP. Misunderstanding and confusion about the schedule, among child caregivers [12] and health workers [14] alike, was among the most common reasons children missed some or all malaria vaccine doses.

Child caregivers’ limited understanding about the number and timing of doses resulted in many missed vaccination visits and, ultimately, missed doses. While especially acute early in the vaccine introduction, for some caregivers the misunderstanding persisted through the fourth dose. Notably, caregivers’ lack of understanding that the child can receive a malaria vaccine dose even if late, was a recurrent finding in the qualitative data. If a caregiver missed the vaccination visit for whatever reason (initial hesitancy, being away from home, or busy with other livelihood and family obligations when a dose was due), many believed it was too late to bring the child. This specific misunderstanding was an important cause of incomplete vaccination and, in some instances, a cause of the child missing malaria vaccine doses altogether.

Health workers were also initially confused about eligibility criteria, and, like child caregivers, some did not know what to do when a child presented late. Situations of caregivers who brought the child late being turned away were reported equally by child caregivers [12] and health workers in all three countries.

With assistance from WHO and technical partners, the EPI programmes deployed several job aids [4] during the MVIP to clarify dose eligibility and improve health worker confidence and adherence to the guidance. The job aids included a mix of health worker training and outreach training and supportive supervision (OTSS) tools. A particularly effective tool was a two-minute educational video developed for use in Ghana and Malawi. The video was shown to health workers from these countries during the R3 interviews and was universally praised as helpful and clarifying, but it had not been seen by most of the health workers interviewed. Explanations about why the video was not widely seen included that it was disseminated only once, that its importance was not emphasized when sent around so may have been neglected, and that the video could not be accessed due to limited data or poor network connection. Other health workers were frustrated by the inability to ask questions while watching the video. For example, upon seeing the video during the R3 interview, one health worker asked:

*“My question is that I have a mother who had a preterm birth, so she didn’t bring her to weighing early. Now the child is eight months old and came to take Penta. I gave her Penta 1 and Penta 2 but* [the educational video shows]* I was supposed to give her the malaria vaccine too. But Penta is given at the same site as the malaria vaccine. I cannot inject the child with both at the same site, so now what should I do?”*

Health worker from Ghana interviewed at R3, reacting to an educational video intended to clarify malaria vaccine eligibility (C3_001)

Although an excellent tool, it was unfortunately under-utilized during the MVIP. It should be noted that these eligibility tools became available late in the introduction [2] and sometimes were not widely disseminated or used.

Despite these factors, at R3 health workers from all three countries remarked that they had a better understanding of the schedule and indicated the value of improved eligibility tools. In Ghana, toward the end of the MVIP, the EPI program made the educational video an integral part of supportive supervision, playing it during these visits to clarify lingering confusion and to provide essential reminders to health workers about the use of recording tools (Barrier 3) and key messages to emphasize with caregivers (Table 2). Positive feedback from health workers and improved adherence to the dosing protocol indicate benefits of using such tools.

### Recommendations to mitigate barriers due to complexity of the schedule (Barrier 2)

Four complementary and overlapping recommendations follow observations discovered in the MVIP’s qualitative findings.

#### Avoid reinventing the wheel—adapt existing tools to local and country contexts

While there is no need to reinvent the proverbial wheel, it will be necessary to improve upon it for use in the local terrain. The WHO malaria vaccine introduction guidance [3] provides many examples and links to tools that will be useful both to countries planning an introduction and to those wishing to improve uptake and coverage of the vaccine.

#### Intensify health worker training and outreach training and supportive supervision on eligibility and interpersonal communication

Health workers must be equipped with clear guidance on what to communicate to caregivers before, during, and after vaccination sessions. Additionally, messaging to caregivers about the importance of administering missed doses at the next visit is recommended. As described above, mastering the vaccination schedule, especially when children present late, was difficult for health workers. Intensive initial training on eligibility criteria and the four-dose schedule is in order, and this can be reinforced through on-the-job refresher training and frequent reminders during routine supervisory visits, staff huddles, and readily accessible technical advice channels (e.g., *I encountered some confusion, so I called the disease control officer...*). Orientations and mentoring of new staff (transfers in) can also include focused attention on eligibility and dose timing.

#### Emphasize messaging to health workers on age range of children eligible for vaccination at the time of introduction

Coupled with simple eligibility messaging to caregivers through various communications channels (see below), it is essential that health workers convey clear instructions to caregivers regarding when to return for subsequent doses. Health workers play a critical role in providing reminders to caregivers, facilitating their adherence to the vaccination schedule. To avoid children being turned away if they present late, it is important that health workers understand the wider windows of time within which doses can be administered according to country protocol. As described above, excellent tools clarifying eligibility are available to facilitate health worker training and OTSS.

#### Emphasize and reinforce simple eligibility messaging for child caregivers

Improving coverage of a four-dose malaria vaccine schedule requires concerted efforts on several fronts to get caregivers in the door for the first dose and to help them get additional doses. Some of the key aspects of a multi-pronged strategy include developing malaria vaccine key messages (Table 2) to inform and educate clients (Table 1). Simple, two-fold messaging for caregivers can be reiterated through multiple communication channels:

- As with other vaccines, children who come late for doses can still receive their vaccine; and
- Ask your healthcare worker when to come for the next dose.

Caregivers who are familiar with these core messages may be more likely to adopt the vaccine and complete the schedule. For dose one, understanding that their child can receive the vaccine even if late may allow caregivers with initial barriers—such as initial awareness gaps, hesitancy to accept another vaccine, or being away from home at the first dose visit—time to overcome the barrier and take the child, rather than assuming it is too late. Similarly for dose four, caregivers who are aware that their child can receive the fourth dose late may be better able to comply with the vaccination schedule while accommodating other life or livelihood demands.

## Barrier #3: Challenges tracking doses for individual children

Quality services and program oversight rely on accurate documentation of vaccine delivery. Several of the immunization data quality challenges described by Scobie et al*.* [20] were apparent in the MVIP’s qualitative evidence, particularly around tracking the doses provided to individual children [14]. For busy health workers, dose tracking proved to be especially difficult when record-keeping tools had not been updated to include the malaria vaccine. Replacing record-keeping tools with each new vaccine introduction is typically not feasible, thus requiring improvised procedures using old tools. Many health workers, however, found improvised strategies for the malaria vaccine difficult to use, mainly due to:

- Inadequate space in home records and registries to record details about the dose given (“*Sometimes you don't even know where to capture the malaria vaccine”*).
- Extra record-keeping steps (“*It’s hard to flip through the book to write the details or to check what the child has already received”*).
- Inconsistent use of interim procedures by different health workers (“*If you don’t have time to check/can’t easily find if a dose was given, we just write that it’s the first dose*”).

Difficult-to-use or ill-adapted interim tools and procedures lead to record keeping errors and data gaps, potentially affecting service delivery (with some children missing needed doses and others receiving extra doses), accuracy of coverage reports, and accuracy of data used for stock management. While malaria vaccine data capture improved over time, especially when updated tools became available, difficulties in record keeping and reporting were a major theme found in health worker interviews across all three rounds. The challenge was apparent as well in qualitative data from child caregivers [12], with several individuals at R3 pointing out conflicts between what they were told at the vaccination visit (e.g., “They said he was finished with all the doses”) and what was recorded in the child health book (e.g., a record of the child receiving only three doses).

### Recommendations to address barriers related to record keeping (Barrier 3)

As described above, problems arise when interim and improvised strategies are ill-understood, are difficult to use, or are inconsistently used by different health workers. Taking a few simple measures can improve record keeping continuity and completeness across vaccinators and immunization sites, even when using improvised tools:

#### Develop user-informed interim tools and procedures

Many record-keeping and dose-tracking weaknesses can be avoided when development of tools and systems are thoroughly informed by end-users (i.e., the vaccinators themselves). Taking the time to pretest and use human-centered design methods [21] to develop the interim tools and procedures could ensure they are practical and effective for health workers to use. In the design process, important specific considerations include:

- Ensuring there is adequate and easy-to-access space to write and retrieve dose details.
- Ensuring the register or recording tool is fit for purpose. This means that if children are meant to receive vaccines in the second or third year of life, registers and tally sheets should cover those ages when children would present.
- Minimizing the number of steps health workers are required to take to determine the number of doses a child has already received or to record details about the dose given at the present visit.
- Simplifying tasks across different tools by either defragmenting or segregating records, whichever makes the record keeping burden easier for health workers. If not already in use, unique identification numbers for children will also aid in this regard.

#### Intensify health worker training and OTSS on record keeping systems

As with training and support needs on eligibility and the schedule, health workers need specific, consistent, and recurrent training and guidance on record keeping. To avoid inconsistent or non-use of interim systems, it is important to specify exact procedures to be used (e.g., where dose details are to be recorded) and to emphasize the need for uniform practice across providers and vaccination sites. For new staff (transfers in), thorough orientation on malaria record keeping is required, ideally accompanied by one-on-one mentoring from seasoned colleagues.

#### Plan for other new vaccine introductions

When it is possible to replace record keeping tools with updated versions that include the malaria vaccine, countries can consider taking advantage of the new production opportunity to include other anticipated or unknown new vaccines. Updated tools can be designed to include open spaces and cells to accommodate new, “TBD” vaccines that are yet to be identified.

#### Encourage and teach caregivers to use the child health book to track malaria vaccine doses given and remaining

Child caregivers who actively participate in tracking the vaccination status of their child may be more likely to adhere to the schedule. Moreover, their efforts and engagement may help to prevent record keeping oversights during busy vaccination clinics. Reflecting COMMVAC “teach skills” interventions (Table 1), there is need for repeated encouragement and education for caregivers to consult and use the child health book to stay abreast of the child’s vaccination status and to seek doses when the child is due. This encouragement and education can be integrated into group health talks but may be most effective in the brief provider-caregiver encounter when a dose is administered.

## Barrier #4: Fourth dose complacency and (in)convenience

During the MVIP, relatively low uptake of the fourth dose was most related to the visit’s timing—around 22–24 months of age [12]. Caregivers often did not take the child for the dose if they simply forgot about the new vaccination visit, were busy with livelihood activities (e.g., farming), were pregnant, had a new infant to care for, or were away from home during the fourth-dose visit. Closely tied to these various convenience barriers, some caregivers conveyed a sense of satisfaction with the effectiveness of three doses, suggesting low perceived need for dose four.

Caregivers in the qualitative sample understood the notion of vaccines “helping” or “strengthening” the body to ward off disease. They similarly understood and appreciated the benefits of a partially protective vaccine and the need to continue use of other prevention while taking their child for vaccination [14]. In other words, caregivers understand and respond to nuanced messaging, boding well for expanding messaging and education about the prolongation of protection with a fourth dose.

### Recommendations to improve uptake of the fourth dose (Barrier 4)

#### Emphasize dose four eligibility messaging for child caregivers and health workers

As described in the section on Barrier #2, it is essential that both child caregivers and health workers understand that children should receive all doses of the malaria vaccine, even if a dose was missed. Clear guidance on the age band for administering dose four is available for health workers. This guidance and consistent use of job aids should be emphasized in health worker training and during supervisory visits. Reflecting COMMVAC’s “inform or educate” purpose (Table 1) and MVIP key messages (Table 2), it may be helpful to focus interventions with caregivers on the benefits of extended protection of the child with the final dose. Such complementary messaging will equip health workers with needed information to manage vaccination schedules efficiently, while caregivers are encouraged to prioritize their child’s vaccinations without the burden of remembering specific age bands.

#### Consider aligning the fourth dose with existing vaccines in the second year of life

Aligning the malaria vaccine fourth dose with other vaccines that are given in the second year of a child's life and that already have high uptake can enhance dose four coverage. Based on learnings from the MVIP, Ghana switched the timing of the fourth dose from 24 to 18 months. When provided at 24 months, the fourth dose had required an extra visit outside the age range of current routine childhood vaccinations in Ghana. Given that WHO recommends flexibility in the dosing schedule to optimize uptake, Ghana switched the fourth dose to 18 months to coincide with other established vaccinations. At 18 months, a child in Ghana can now receive a fourth malaria vaccine dose, second measles-rubella dose, and MenA vaccination. Caregivers can also receive ITNs during this visit. The change in the schedule was meant to improve coverage of the fourth dose and save time for caregivers and health workers.

#### Develop systems to identify and reach out to default-vulnerable caregivers for the final dose

As noted above, caregivers in late pregnancy or with new infants to care for are vulnerable to defaulting on dose four in the four-dose schedule. These default-vulnerable caregivers can be identifiable through maternal and child health records and can be targeted (preemptively or once flagged as “late” for the fourth dose) for COMMVAC “remind and recall” and “provide support” interventions (Table 1) with personalized outreach through phone calls or a home visit and messaging. Reiterating protective benefits of a four-dose schedule may be sufficiently persuasive. As needed and appropriate, health workers may prompt a discussion about alternative ways to ensure the child is vaccinated. For instance:

- Informing or reminding the caregiver of an upcoming outreach service that will be closer to the caregiver’s home.
- If the caregiver is unable to bring the child to services, suggesting or brainstorming alternative options, such as a relative, friend, or neighbour bringing the child instead.
- Exploring the possibility of aligning the fourth-dose vaccination visit with an upcoming visit for other vaccines or other health services, including antenatal or post-natal care appointments.

Routinely maintaining up-to-date contacts for the caregiver will be important for such personalized outreach and messaging. If possible, at each under-five clinic, caregivers can be asked to verify and update their contact information.

#### Intensify persuasive messaging to caregivers about prolonging protection with a fourth dose

Malaria vaccine benefits were perceived early in the introduction and by virtually all caregivers in the HUS sample, regardless of the malaria vaccination status of their child [12]. While perceived benefits of the vaccine promoted trust and uptake, at the same time it possibly dissuaded some to take the child for the fourth dose, reflected in such comments as:

*“I think three doses are good enough.” “The truth is, I don’t plan on taking her; I don’t have time.”*

More nuanced or additional “inform and educate” and “remind and recall” (Table 1) messaging about the specific and added value of the fourth dose is needed for caregivers who become complacent or who do not perceive need for a fourth dose. Intensified messaging about fourth-dose benefits will increase caregivers’ understanding of why a dose is recommended late in the second year of life, while also promoting adherence to the schedule.

## Barrier #5: Subnational implementation challenges

Especially in contexts where malaria is widespread and demand for a vaccine is high, there are distinct programmatic and operational challenges with subnational and phased introductions of the malaria vaccine. Qualitative findings from the MVIP [12, 14] indicate a risk that community members may view subnational provision of the malaria vaccine with suspicion, suspecting socio-political favoritism behind decisions about which communities are receiving or not receiving the vaccine. At the front lines of service delivery, health workers must manage community expectations and potentially unmet demand (e.g., *“a rush for the vaccine”* from people coming from outside the vaccinating area). Health workers additionally face significant operational challenges—for both service delivery and record keeping—in managing situations of children from non-vaccination areas coming to vaccinating clinics and, conversely, of children from vaccination areas travelling away from home when a dose is due. Both these situations can result in incomplete vaccination.

### Recommendations to address challenges with a subnational introduction (Barrier 5)

As mentioned in the section on Barrier #1 above, in announcing arrival of the malaria vaccine or any other malaria intervention intended for use in parts of the country, it is critical to include information about initial subnational delivery, the idea of a package of interventions, and national scale-up plans. Engaging stakeholders early and preparing health workers are additionally recommended, reflecting COMMVAC “enhance community ownership” interventions (Table 1).

Engaging diverse stakeholders early in the planning of a malaria vaccine introduction serves three main purposes:

- *Inform*. It first presents an opportunity to inform the stakeholders about the malaria vaccine, including its benefits as well as the need to continue other malaria prevention behaviours with vaccinated children. Sharing information about EPI introduction plans is also important, particularly details about subnational and phased introductions.
- *Listen*. Carefully selected stakeholders can bring important insights and experience to the malaria vaccine introduction team, flagging potential barriers to acceptance and effective delivery. For subnational introductions, it will be critical to engage stakeholders, including those from the malaria sector, in discussions about criteria for selecting malaria vaccine introduction sites. These discussions need to address a mix of considerations, such as equity, fairness, epidemiology, and social perceptions.
- *Collaborate.* Stakeholders who are well informed about the malaria vaccine and introduction plans can foster community connections and trust, helping to pave the way for a successful launch and uptake of the malaria vaccine.

#### Provide clear instructions to both non-vaccinating and vaccinating sites

Whether or not they are providing the malaria vaccine, staff and community health workers from all health districts and facilities would benefit from basic orientation about the vaccine’s introduction and, at a minimum, about simple messages about eligibility. Focus can be given to non-vaccinating and vaccinating districts/facilities adjacent to one another, involving development of complementary and coordinated systems to serve caregivers who frequent under-five services in both areas. Such a coordinated approach is especially important in contexts with seasonal migration or with large migrant populations, and may involve:

- *Non-vaccinating services*. Health workers at non-vaccinating sites can be equipped to receive a child from a vaccinating site who is due for a malaria vaccine dose. Instructions may include:
  - Contacting the caregiver’s home clinic—the vaccinating site—to make them aware that the child was received at the non-vaccinating site and will be late for the malaria vaccine dose.
  - Flagging the child’s health card “due for the malaria vaccine” to remind the caregiver to take the child once back home and to call attention to the needed dose for health workers at the home clinic.
  - Reminding and encouraging the caregiver to take the child for the malaria vaccine even though s/he will be late for the dose.
  - Utilizing special identifiers on the homebased records of children coming from vaccinating areas to facilitate easy differentiation from those arriving from non-vaccinating areas.
- *Vaccinating services*. Conversely, health workers at vaccinating sites need clear instructions on what to do if a child from a non-vaccinating area is brought to their clinics. Without such guidance, health workers will establish their own protocols and procedures that may not align with national guidance (e.g., *“If we think the schedule will be a major problem due to migration, we don’t start at all”*).

## Barrier #6: New vaccine hesitancy

As with any new vaccine introduction, child caregivers interviewed for the MVIP’s qualitative study had varying degrees of apprehension about their child receiving another vaccine, additional injections, or both. Most caregivers in the sample, however, accepted initial malaria vaccine doses without hesitation or despite some initial trepidation. A notable minority of individuals (caregivers themselves or their partners) either refused the malaria vaccine, typically due to past negative experiences with a vaccination by injection, or delayed uptake of the first dose by several months allowing them time to gain confidence in its safety [12]. Some delayed accepters who took their child late to receive the first dose were turned away by health providers, even though the child was still eligible to receive the first dose (see Barrier #2).

### Recommendations to address new vaccine hesitancy (Barrier 6)

Effective stakeholder engagement and wide and recurrent dissemination of key “inform and educate” messages (Table 2) will avert many refusal and defaulter cases. Additional efforts are needed when the caregiver’s hesitancy derives from a past negative experience with vaccination, whether the incident qualifies as an actual adverse event following immunization, or if it is perceived by the caregiver to be linked to vaccination.

#### Establish systems to flag refusal/default-vulnerable caregivers for follow-up

Caregivers who return to the clinic or complain about adverse effects from vaccination—perceived or real—can be flagged to monitor the vaccination status of their children and for follow-up if found to be late for a visit. At the same time, it is important to acknowledge the practical challenges of implementing such activities and adding them to the health workers’ already heavy workload. For countries wanting to develop systems to flag and follow-up with default-vulnerable caregivers, it will be important to engage health workers in the design and pretesting of any procedures and tools developed to support the activity. Human-centred design, which involves end users (in this case, health workers) in the creation and testing process, is likely to be the most efficient way to develop effective tools to address this challenge [21].

#### Support health workers to provide personalized outreach and education to refusal/default-vulnerable caregivers

An important COMMVAC “provide support” intervention (Table 1), personalized education for refusal/default-vulnerable caregivers, is best done by experienced and knowledgeable health workers who can address a variety of issues that may be the source of hesitancy. Basic techniques for counseling can be integrated into formal training, if possible, and reinforced through OTSS. It is important to acknowledge that this would represent a major shift in practice for many health workers in busy and resource-constrained clinics. Specific training and support will be necessary if the MOH commits to implementing this type of personalized outreach. Essential elements of personalized interpersonal communication may include:

- *Eliciting the core cause(s) of hesitancy*—For example, health workers prompting the caregiver: *Explain to me everything that happened that caused you to be concerned.*
- *Responding to the caregiver’s specific concerns*. It would be helpful if health workers were prepared to address and document caregivers’ specific questions or concerns following immunization. It is important that health workers educate caregivers about the process of reporting any symptoms or effects, regardless of the perceived severity, as part of the protocol for reporting adverse events. Providing caregivers with easy ways to report symptoms, whether during door-to-door visits or by using SMS and call centers, would also be important. While the relationship of an event to the vaccine is determined by expert committees, immediate management of all complaints can be conducted at the facility level. Following this, reports would be escalated to the next level for further investigation and action as per protocol.
- *Persuading the caregiver to bring the child for vaccination*. In addition to emphasizing the benefits of protecting the child from malaria, other persuasive techniques may include describing steps that will be taken to ensure safe delivery (e.g., arrangements for the vaccine to be administered by an experienced provider) and discussing easier ways for the caregiver to bring the child (e.g., to an outreach service that is closer to her home).

Instituting a practice at every childhood health service to check accuracy of the caregiver’s contact information and update the information if needed will facilitate personalized outreach to refusal/default-vulnerable caregivers.

#### ***Develop a comprehensive risk communication plan ***[22]*** to mitigate refusal and defaulter cases***

A comprehensive risk plan would include stakeholder engagement, wide and recurrent dissemination of and training in the use of key messages, and a feedback and rumor tracking system to monitor and respond to community feedback, concerns, perceptions, and misinformation. It would be important to adapt communication and informational materials in response to feedback and perceptions. To complement this activity, it would help to train key actors to use interpersonal communication and dialogue-based interventions to communicate with communities about malaria vaccination.

## Barrier #7: Service interruptions

During the MVIP, vaccination services became temporarily unavailable for a variety of reasons. COVID closures were the most frequent cause of service interruptions, but other interruptions were caused by unannounced changes to facility schedules for vaccination visits, health worker strikes, and stockouts [12, 14]. Several children missed some or all malaria vaccine doses due to these interruptions when caregivers were demotivated after one or more failed attempts to receive service or were unaware of service resumption. Service interruptions will inevitably occur and may be unavoidable. A mix of communication and systems strengthening steps, however, can minimize missed vaccinations due to an interruption in health services.

### Recommendations to minimize missed doses due to service interruptions (Barrier 7)

#### Ensure the community is aware of service interruptions and service resumption

Caregivers are often unaware of general service cancellations or of temporary disruption of a specific service, such as delivery of the malaria vaccine. Use of a special kind of COMMVAC “inform and educate” (Table 1) intervention can be helpful whenever community members need to be informed in advance of service closures. It would be equally important to let the community know when the service has been restored. In such service resumption announcements, reiterating the key message to bring the child for vaccination, would be key, even if it the child receives the dose(s) late. Making these public announcements through community-based health workers, local leaders, and local media will further serve to “enhance community ownership” (Table 1) and partnership with local health services.

#### Perform regular audits to improve accuracy of vaccine distribution

To mitigate stockouts, health facilities and district vaccine stores can conduct routine supervision and data quality audits to ensure accuracy of reported data. Regular forecasting at both national and subnational levels will help to anticipate demand accurately and ensure vaccine supplies are appropriately restocked. Often, estimates come from unreliable reports, leading to over- or under-estimation of vaccine needs. Addressing this issue requires applying accurate targeted population figures for quantification, as many countries face challenges with their denominator calculations. Routine updates of stock levels and consumption data will help minimize count errors and improve the accuracy of stock distribution.

#### Strengthen subnational supply management and distribution systems

At the regional and district levels, similar processes are in place to ensure effective distribution and management of vaccine supplies. This includes maintaining up-to-date inventory records with appropriate reconciliation between physical stocks in the vaccine fridges and stock ledger records. Adequate lead time should be allowed for the delivery of vaccines and devices to prevent shortages. Additionally, it is important to avoid maldistribution of available stocks, especially for antigens that are administered alongside the malaria vaccine.

## Conclusion

Adding a four-dose malaria vaccine to an already crowded routine immunization schedule presents several challenges, as seen during the 2019–2023 MVIP in Ghana, Kenya, and Malawi. Nonetheless, the three pilot countries reached coverage levels at or higher than expected of the first three doses during the MVIP, and impact was high despite low uptake of dose four [18]. The MVIP experience and several studies have provided a rich set of guidance and tools to support both malaria vaccine expansion in MVIP countries and introduction of the vaccine in other countries. This paper highlights strategies to address the complexity of the schedule, the need to track four doses for individual children, subnational malaria vaccine implementation, new vaccine hesitancy, service disruptions, and other barriers to vaccine delivery and uptake (see Table 3 below for summary). The observations and recommendations detailed in this paper are intended as a complement to the wide range of materials, guidance and tools available elsewhere.

**Table 3:** Summary of Barriers and Recommendations to Improve Uptake and Completion of a Four-dose Malaria Vaccine Schedule Based on the MVIP’s Qualitative Evidence

Main barriers		Key recommendations	
1	Communication gaps and needs. Most of the reasons malaria vaccine doses were missed had a communication and demand promotion element, typically betraying a critical need for more information, persuasion, or support	1.1	Communicate to Vaccinate—Adopt or reinforce a systematic and comprehensive approach to vaccination communication
		1.2	Announce the malaria vaccine with a *wide* and *loud* launch
2	Complexity of the schedule. Misunderstanding and confusion about the schedule, among child caregivers and health workers alike, were among the most common reasons children missed some or all malaria vaccine doses	2.1	Avoid reinventing the wheel—Adapt existing tools to local and country contexts
		2.2	Intensify health worker training and OTSS on eligibility and interpersonal communication
		2.3	Emphasize messaging to health workers on age range of children eligible for vaccination at the time of introduction
		2.4	Emphasize and reinforce simple eligibility messaging for child caregivers
3	Challenges tracking doses for individual children. Though essential for providing a quality service and for program oversight, accurate documentation of malaria vaccine doses delivered was a significant challenge for health workers, sometimes leading to children missing doses or given extra doses	3.1	Develop user-informed interim tools and procedures
		3.2	Intensify health worker training and OTSS on record keeping systems
		3.3	Plan for other new vaccine introductions
		3.4	Encourage and teach caregivers to use the child health book
4	Fourth dose complacency and (in)convenience. The relatively low uptake of the fourth dose was most related to the visit’s timing late in the child's second year of life. Additionally, some caregivers conveyed a sense of satisfaction with the effectiveness of three doses, suggesting low perceived need for dose four	4.1	Emphasize dose four eligibility messaging for child caregivers and health workers
		4.2	Consider aligning the fourth dose with established vaccines in the second year of life
		4.3	Develop systems to identify and reach out to default-vulnerable caregivers for the final dose
		4.4	Intensify persuasive messaging to caregivers about prolonging protection with a fourth dose
5	Subnational implementation challenges. In contexts where malaria is widespread and demand for a vaccine is high, there are distinct programmatic and operational challenges with subnational and phased introductions of the malaria vaccine, including public perceptions of favoritism, a potential "rush on the vaccine" at vaccine delivery sites, and burden on the health system to maintain accurate records for caregivers who frequent health services in both vaccinating and non-vaccinating areas	5.1	Engage stakeholders early and comprehensively
		5.2	Provide clear instructions to both non-vaccinating and vaccinating sites
6	New vaccine hesitancy. A notable minority of individuals either refused the malaria vaccine, typically due to past negative experiences with a vaccination by injection, or delayed uptake of the first dose by several months allowing them time to gain confidence in its safety	6.1	Establish systems to flag refusal/default-vulnerable caregivers for follow-up
		6.2	Support health workers to provide personalized counseling to refusal/default-vulnerable caregivers
		6.3	Develop a comprehensive risk communication plan to mitigate refusal and defaulter cases
7	Service interruptions. Several children missed some or all malaria vaccine doses during the MVIP due to various service interruptions when caregivers were demotivated after one or more failed attempts to receive service or were unaware of service resumption	7.1	Ensure the community is aware of service interruptions and service resumption
		7.2	Perform regular audits to improve accuracy of vaccine distribution
		7.3	Strengthen subnational supply management and distribution systems

## Data Availability

No datasets were generated or analysed during the current study.
